# Ticagrelor and Statins: Dangerous Liaisons?

**DOI:** 10.1007/s10557-024-07624-7

**Published:** 2024-09-30

**Authors:** Bianca Rocca, Elisabetta Bigagli, Elisabetta Cerbai

**Affiliations:** 1 Department of Safety and Bioethics, Catholic University, Largo F. Vito 1, Rome, Italy; 2 Department of Medicine and Surgery, LUM University, SS 100, km 18, Casamassima, Bari, Italy; 3 Department of Neurosciences, Psychology, Drug Research and Child Health (NEUROFARBA), University of Florence, Viale Pieraccini 6, Florence, Italy

**Keywords:** Ticagrelor, Statins, Drug interactions, Cytochrome P450, Rhabdomyolysis, Transporters

## Abstract

Polypharmacy is often necessary in complex, chronic, comorbid and cardiovascular patients and is a known risk factor for potential drug-drug interaction (DDI) that can cause adverse reactions (toxicity or therapeutic failure). Anti-thrombotic drugs (largely low-dose aspirin and a platelet P2Y12 receptor inhibitor) and statins are among the most co-administered drugs in cardiovascular patients. Ticagrelor is a selective antagonist of the platelet P2Y12-receptor, highly effective in inhibiting platelet aggregation and bio-transformed by the CYP3A4 and substrate of transporters, such as the breast cancer resistance protein (BCRP). Statins have different pharmacokinetic profiles; some undergo CYP3A4-mediated metabolism; rosuvastatin is primarily metabolized by the CYP2C9; and they have different affinities for drug transporters. Rhabdomyolysis is a very rare but severe adverse event, which is specific for statins which can be triggered by DDIs that increase statin’s concentrations through blockade of their biotransformation and/or elimination. Large pharmacovigilance and small observational studies reported increased rhabdomyolysis in patients treated with some statins and ticagrelor but not aspirin, clopidogrel or prasugrel. Recent studies in vitro, pharmacokinetic trials and in silico drug modelling identified and validated the BCRP inhibition by ticagrelor, as a mechanism contributing to the DDI with statins, as ‘victim’ drugs, leading to increased rhabdomyolysis. While the clinical impact of this DDI deserves further investigation, a careful evaluation should be advised when ticagrelor is co-prescribed with some statins.

## Polypharmacy and atherothrombotic diseases

Polypharmacy is broadly defined as the *concurrent use of multiple medications*, and it is often referred to as the routine use of ‘five or more’ medications, including over-the-counter, prescribed traditional and complementary medicines [1]. However, there are no consensus-specific definitions(s) in relation to different patient’s ages and clinical settings. Polypharmacy is often necessary in complex, chronic and comorbid patients. However, polypharmacy can be dangerous when it comprises drugs that are either unnecessary and may lower drug’s adherence and persistence or, even worse, that may interact and reduce the safety and efficacy of the therapy, due to specific pharmacodynamics and/or pharmacokinetics profiles. Indeed, polypharmacy is a known risk factor for potential drug-drug interaction (pDDI), which, only if clinically relevant, may ultimately lead to an overt adverse reaction (toxicity or therapeutic failure), with a negative impact on the patient’s quality of life, may cause disabling conditions, mortality as well as impact on health system costs due to (re)hospitalization or long-term complications. Various online databases are available to screen for pDDIs, considering that not all pDDIs have clinically relevant consequences. Lexi-interactions and Micromedex have been proven as the most sensitive, e.g. able to detect the pDDIs of clinical relevance, and specific, e.g. ignoring pDDIs that are clinically negligible [2]. The Lexicomp grade interactions from A (of no clinical concern) to D (evidence for interaction in a clinically significant manner with a suggestion to modify regimen), and the X category identifies a clear contraindication to the co-medication due to risks that exceed benefits [2].

Patients living with cardiovascular diseases (CVDs) are particularly exposed to polypharmacy, pDDIs and their clinical effects [3]. In the apixaban versus warfarin in patients with atrial fibrillation (ARISTOTLE) phase III trial, patients with atrial fibrillation at study entry had a median of 6 prescribed drugs and a documented polypharmacy (≥ 5 co-prescribed drugs) in ~ 77% of the trial population [4]. In the same trial, the number of co-prescribed drugs was significantly associated with a progressive loss of the superior safety of apixaban versus warfarin. In a large, record-based study, patients hospitalized for acute coronary syndrome (ACS) received a mean of 9.9 ± 2.6 drugs per day, whose Lexicomp categories C, D and X were documented in 75.3%, 4.8% and 0.3% of the patients, respectively [5]. The most represented drugs in these patients were, in decreasing order, anti-thrombotic drugs (largely aspirin and a P2Y_12_ receptor inhibitor), statins, blood-pressure-lowering, glucose-lowering drugs and benzodiazepines. Thus, polypharmacy is nowadays a relatively ‘standard’ condition in the majority of CVD patients, and the pDDIs are rather frequent.

## Pharmacokinetics of Ticagrelor

Ticagrelor is a complex carbocyclic nucleoside analogue and a reversible, selective antagonist of the platelet P2Y_12_-receptor. It is highly effective in inhibiting ADP-dependent platelet aggregation and in contrast to clopidogrel and prasugrel, it is not a prodrug. Ticagrelor is rapidly absorbed, within 1.3–3 h, and is mainly bio-transformed by the CYP3A4 leading also to an equipotent active metabolite (AM) AR-C124910XX (approx. 1/3 of the absorbed dose, Table 1). One‐third of ticagrelor metabolites are excreted in urine and two‐thirds in faeces with an elimination half-life of ~ 7 h for the parent drug and of ~ 9 h for its AM. Both ticagrelor and its AM are substrates and weak inhibitors of the P-glycoprotein (P-gp) efflux transporter. Ticagrelor also inhibits the organic anion–transporting polypeptides (OATP) 1B1, 1B3 and 2B1, but this interaction seems with no clinical relevance because of the low portal vein concentrations. By contrast, ticagrelor significantly inhibits breast cancer resistance protein (BCRP), thereby raising the plasma concentrations of other BCRP substrate drugs [6].

**Table 1:** Pharmacokinetic properties of ticagrelor, statins, bempedoic acid and ezetimibe [6–11]

	*Mechanism of action*	*Prodrug*	*Bioavailability* %	*Active metabolite(s)*	*T*_max_ *hours*	*Half-life hours*	*Protein binding* %	*Transporters*	*Major enzymes*	*Faecal excretion* %	*Renal excretion* %
*Ticagrelor*	Antagonist of platelet P2Y_12_-receptor	No	~ 36	AR-C124910XX	1.3–2 (Ticagrelor) 1.5–3 (AR-C124910XX)	~ 7 (Ticagrelor) ~ 9 (AR-C124910XX)	> 99	P-gp, BCRP, OATP1B1, 1B3, 2B1	CYP3A4, CYP3A5, CYP2C9	58	27
*Atorvastatin*	Inhibitor of HMG-CoA reductase	No	14	Hydroxy-atorvastatin	1–2	14	≥ 98	P-gp, ,NTCP OATP1B1, 1A2, BCRP	CYP3A4	70	< 2
*Lovastatin*	Inhibitor of HMG-CoA reductase	Yes	< 5	β-Hydroxyacid-lovastatin	2–4	2–3	> 95	OATP1B3, 2B1, BCRP, NTCP	CYP3A4	83	10
*Pravastatin*	Inhibitor of HMG-CoA reductase	No	17	No	1–1.5	1.8	50	P-gp, MRP2,NTCP, OATP1B1, 1B3, 1A2, 2B1, BCRP	Sulfonation	71	20
*Rosuvastatin*	Inhibitor of HMG-CoA reductase	No	20	Minimal	3–5	19	88	OATP1B1, 1B3,1A2 NTCP, BCRP	CYP2C9, CYP2C19 (minor)	90	10
*Simvastatin*	Inhibitor of HMG-CoA reductase	Yes	< 5	β-Hydroxyacid-simvastatin	4	2	95	P-gp, NTCP OATP1B1, 2B1, BCRP	CYP3A4	58	13
*Bempedoic acid*	Inhibitor of ATP-citrate lyase	Yes	90	ETC-1002-CoA ESP15228-CoA	3.5	15–24	99	OATP1B1, 1B3	Glucuronidation	30	70
*Ezetimibe*	Inhibitor of NPC1L1	No	Variable (35–60%)	Ezetimibe-glucuronide	4–12 (Ezetimibe) 1–2 (Ezetimibe glucuronide)	24	99.7	OAT1B1, 1B3, P-gp, MRP2	Glucuronidation	90	10

*HMG-CoA* hydroxy-3-methylglutaryl coenzyme A, *NPC1L1* Niemann–Pick C1-like 1 protein, *CYP* cytochrome P 450, *BCRP* breast cancer resistance protein, *OATP* organic anion transporting polypeptides, *P-gp* P-glycoprotein, *MRP2* multidrug resistance protein 2, and *NTCP* sodium/taurocholate co-transporting polypeptide

## Pharmacokinetics of Statins

The mechanism of action and the pharmacokinetics of statins are summarized in Table 1. All statins selectively inhibit HMG-CoA reductase and do not have relevant pharmacodynamic-based interactions. However, different statins differ in their chemical structure, lipophilicity and pharmacokinetic profiles. Lovastatin and simvastatin are lactone pro-drugs, enzymatically hydrolyzed to their active hydroxy-acid forms, whereas the other statins are administered as active forms (Table 1). All statins are rapidly absorbed following oral administration and undergo extensive hepatic first-pass metabolism which accounts for their low systemic bioavailability. Simvastatin, atorvastatin and lovastatin undergo CYP3A4-mediated metabolism, while pravastatin is metabolized by sulfation. Rosuvastatin has limited CYP450-dependent metabolism, primarily by CYP2C9 and to a lesser extent by CYPC19. Statins are mainly eliminated through the bile with overall modest renal excretion and half-lives ranging from < 5 h (lovastatin, pravastatin and simvastatin) to 14 h (atorvastatin) and 19 h (rosuvastatin) [7].

Statins also show different affinities for membrane drug transporters involved in their absorption and efflux in the intestine, liver and kidney. The hepatic uptake of all statins is largely dependent on OATPB1 and OATP1B3, but other transporters such as P-gp, BCRP, multiple drug resistance protein 2 (MRP2) and sodium-dependent taurocholate co-transporting polypeptide (NTCP) may also bind statins [8]. The systemic exposure and clearance of statins may be influenced by the competition of other drugs for these transporters and this mechanism may explain pDDIs [8].

## Pharmacokinetics of Other Lipid-Lowering Drugs: Bempedoic Acid and Ezetimibe

The mechanism of action and the pharmacokinetics of bempedoic acid and ezetimibe are summarized in Table 1. Bempedoic acid is a pro-drug that undergoes conversion into its AM, the bempedoyl-CoA, by the very long chain acyl-CoA synthetase-1 (ACSVL1), primarily expressed in the liver but not in the skeletal muscle, which may limit the potential myotoxic effects. The inhibition of the hepatic ATP citrate lyase (ACLY) by bempedoyl-CoA suppresses cholesterol synthesis and triggers LDL receptor upregulation and LDL uptake from the peripheral blood, thereby decreasing circulating levels. Bempedoic acid is rapidly and highly absorbed through the small intestine, is plasma protein bound by ~ 99%, and its metabolism occurs mainly via UDP-glucuronosyltransferase-2B7 (Table 1), thus bempedoic acid and its AM are not metabolized by the CYP450 enzymes or substrates of BCRP. However, bempedoic acid and its glucuronides are weak inhibitors of OATP1B1 and OATP1B3 and co-administration with some statins may increase the concentrations due to pDDIs at the level of these transporters [9]. In fact, its concomitant use with high dose simvastatin (> 40 mg) is contraindicated [10]. Bempedoic acid is primarily eliminated in urines (70%).

Ezetimibe inhibits the absorption of cholesterol by blocking the Niemann-Pick C1-like 1 (NPCL1) protein in the small intestine. Following oral administration, it is rapidly absorbed and extensively metabolized (80%) to the ezetimibe-glucuronide AM, with minor CYP450-mediated metabolism (Table 1); once glucuronated, ezetimibe and its AM undergo extensive enterohepatic recycling and then are slowly excreted mainly in the faeces (90%) [11]. Ezetimibe and its AM are substrates of the intestinal efflux transporters MRP2 and P-gp [11].

## Clinically-Relevant Interactions Between Ticagrelor and Statins

In patients with ACS, dual anti-platelet therapy (DAPT) with aspirin and a P2Y_12_ receptor inhibitor, either ticagrelor or prasugrel, is the first-line treatment in the 12 months after the acute event [12]. Moreover, tight LDL cholesterol control is strongly recommended in those patients at very high cardiovascular risk, contributing to reduce the recurrence of fatal and non-fatal atherothrombotic events [12]. In the ticagrelor versus clopidogrel in patients with acute coronary syndromes (PLATO) phase III trial, which compared aspirin with clopidogrel to aspirin with ticagrelor post-ACS, ~ 90% of the participants at trial entry were treated with a statin [13]. In particular, since there is a direct, continuous correlation between LDL levels and the risk of a major atherothrombotic event, a statin administered at a high dose is highly effective in maximally reducing LDL cholesterol, and therefore it is recommended for patients at very high CV risk [12]. More intensive statin regimens cause a significant additional 15% relative reduction (95% *CI* 11–18; *p* < 0.0001) in major vascular events (coronary death, non-fatal MI, coronary revascularisation and ischaemic stroke), as compared to less intensive regimens [14]. Rhabdomyolysis is a very rare but severe adverse event, specific for statins, estimated to be 4 (standard error-SE 2) per 10,000 patients in trials using high-dose statin and 1 (SE 1) per 10,000 patients in trials using standard statin dosing [14]. Aside from the high doses, other risk factors for rhabdomyolysis are age, diabetes, renal impairment and those drug-drug interactions (DDIs) which increase statin’s concentrations through blockade of their biotransformation and/or elimination [15].

By using the World Health Organization (WHO) pharmacovigilance database (VigiBase®), Roule and colleagues studied 9489 reports of rhabdomyolysis in patients treated with a statin, focusing on 2464 reports (26%) in patients co-treated with anti-platelet drug(s), e.g. aspirin and/or a P2Y_12_ inhibitor (prasugrel, ticagrelor and clopidogrel). As compared to a statin alone as the reference group, rhabdomyolysis was significantly increased by co-administration of ticagrelor (either alone or as part of DAPT) and atorvastatin (adjusted reporting odds ratio-*ROR* 1.30 [1.02–1.65]) or rosuvastatin (adjusted *ROR* 1.90 [1.42–2.54]), and there was a trend also for simvastatin (adjusted *ROR* 1.42 [0.92–2.18]). Unlike ticagrelor, when the same statins were co-administered with aspirin, clopidogrel (single or as part of the DAPT) or prasugrel (single or as part of the DAPT) rhabdomyolysis reports were similar to the statin alone [16]. Furthermore, in the same study, the risk of rhabdomyolysis in ticagrelor-treated patients increased with age ≥ 75 years, chronic kidney disease (*ROR* 7.15 [3.87–13.20] and statin dose [16]. Since ticagrelor approval and before this pharmacovigilance study, sporadic reports had described rhabdomyolysis in patients on a statin and ticagrelor [17]. A very recent observational study in 936 ticagrelor-treated patients followed for 1 year post-ACS, reported 4 cases (2 fatal and 2 successfully treated) of rhabdomyolysis in patients on high-dose statins, i.e. 40 mg rosuvastatin (*n* = 3) or 80 mg atorvastatin (*n* = 1) [18].

The initial reports hypothesized that this severe, clinically relevant DDI between ticagrelor and at least atorvastatin and simvastatin was due to the inhibition of ticagrelor (as perpetrator drug) to the CYP3A4-dependent statin biotransformation, thus leading to an increased concentration and risk of clinically-relevant DDIs, including rhabdomyolysis. Consistently, the co-administration of ticagrelor, atorvastatin or simvastatin and strong inhibitors of the CYP3A4 (e.g. ketoconazole) is contraindicated by regulatory agencies. In particular, ketoconazole increases the area under the curve (*AUC*) of ticagrelor by 7.3-fold, of simvastatin by ~ 5 folds and of rosuvastatin by ~ 2 folds [19]. Based on limited data and a less intense effect of cyclosporin as a probe drug for P-gp interactions on the *AUC* and *C*_max_ of ticagrelor, the EMA discourages the association with drugs that are potent P-gp and moderate CYP3A4 inhibitors (e.g. verapamil and quinidine) that also may increase ticagrelor exposure, advising ‘caution’ if the association cannot be avoided. However, neither statins nor ticagrelor are classified as strong inhibitors of either CYP3A4 enzyme and/or P-gp transporter. Hence, these mechanisms of pDDIs cannot fully explain the pharmacovigilance data and the severity of DDIs associated with co-administration of some of the most used statins and ticagrelor.

An initial study investigated the pharmacokinetic interaction between ticagrelor (90 mg bid) and a single, high-dose of atorvastatin or simvastatin (80 mg) in healthy volunteers. The blood concentrations of ticagrelor and of its AM were unaffected by the co-administration of either atorvastatin or simvastatin. Conversely, in the presence of ticagrelor co-administration, the concentration of atorvastatin increased by ~ 30% with no change in elimination half-life, and the concentrations of simvastatin increased by ~ 50%. These effects led to classify ticagrelor as a weak CYP3A4 inhibitor and as the ‘perpetrator’ drug in relation to atorvastatin and simvastatin as the ‘victim’ drugs, although this CYP3A4-based DDI was considered minor. Moreover, rosuvastatin is not substrate of the CYP3A4, but it is biotransformed by the CYP2C9, and ticagrelor is not a substrate for CYP2C9, thus leaving this interaction pharmacologically unexplained.

**Fig. 1:**
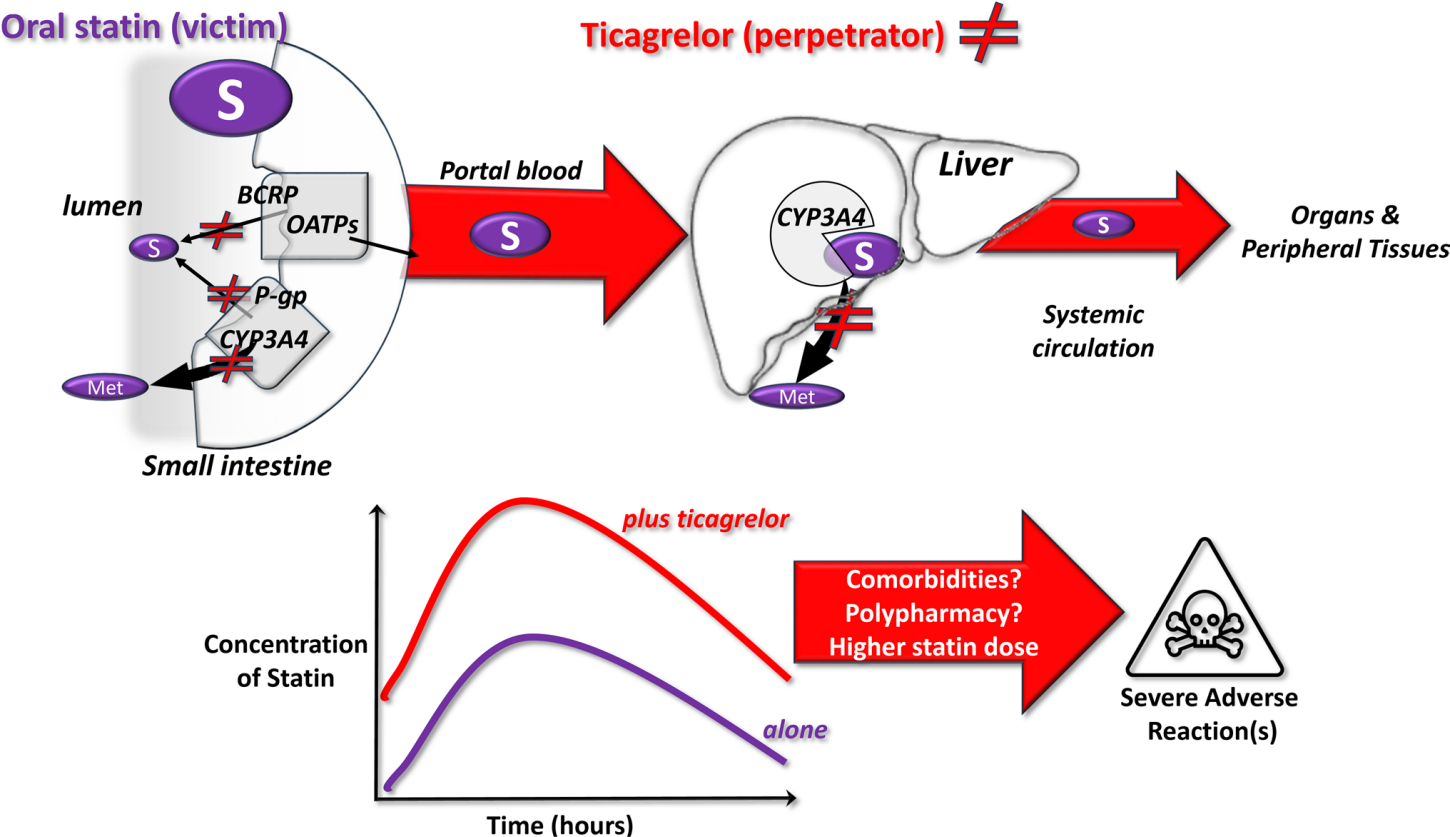
Possible mechanisms underlying the clinically relevant interactions between ticagrelor as perpetrator drug and some statins as victim drugs. Upper panels: pathways of statin metabolism from the small intestine to the liver via the portal blood and to the downstream tissues and organs via the systemic circulation; ticagrelor inhibits the membrane transporters BCRP and P-gp (statin efflux/metabolism) in the enterocytes on  the gut lumen and the biotransforming CYP3A4 enzyme, thus blocking some steps of the elimination and biotransformation of statins. Lower panels: as a consequence of a reduced elimination, the co-administration of ticagrelor with some statins leads to increased plasma levels up to 2-folds of the statins and to potential severe DDIs; comorbidities (e.g. renal failure and ageing), chronic/occasional polypharmacy (e.g. gemfibrozil, anti-hypertensive or anti-microbic agents) and the statin daily dose can concur to cause severe adverse reactions. Abbreviations: BCRP, breast cancer resistance protein; CYP, cytochrome; Met, metabolite(s); P-gp, P-glycoprotein; and OATP, organic anion–transporting polypeptides

Recently, Lehtisalo and colleagues provided new data and identified other pharmacokinetic pathways influenced by ticagrelor, which could explain the clinically relevant DDI with rosuvastatin leading to increased rhabdomyolysis risk. Initial in vitro studies showed that ticagrelor inhibits the transporters BCRP, OATP1B1, -1B3 and -2B1 at concentrations in the low μM range, consistent with those reached at the level of the intestine mucosa, before entering the portal blood (Fig. 1). This would slow the extrusion of rosuvastatin. An in silico model of ticagrelor predicted an increase in rosuvastatin plasma concentration by over 2 folds via the inhibition of the BCRP [6]. This model was then validated in vivo in humans in a placebo-controlled, crossover study in healthy subjects [6]. When subjects were given ticagrelor and rosuvastatin, both the area under the curve (*AUC*) and the maximal concentration (*C*_max_) of rosuvastatin significantly increased by 2.6-fold (90% confidence intervals 1.8–3.8 and 1.7–4.0, respectively), and rosuvastatin’s half-life was almost doubled as compared to rosuvastatin + placebo. By using selective substrates of the OATPs, this study suggested that BCRP inhibition by ticagrelor at the intestinal level, before the first liver passage, is the probable mechanism that accounts for the nearly threefold increase in rosuvastatin’s concentrations [6]. Importantly, this study was performed in healthy subjects, hence in the absence of polypharmacy or comorbidities. In patients, this increased exposure may be more variable and reach higher levels due to additional factors such as comorbidities, age and co-medications. The possible mechanisms underlying clinically-relevant DDI between ticagrelor and statins are depicted in Fig. 1.

Interestingly, in the PLATO study, ticagrelor was also associated with an increase in serum creatinine and to a decrease in renal function, as compared to clopidogrel, mostly in the elderly [13]. Although the kidney is not a major excretion route for statins, this can also contribute to increased statin concentrations, triggering clinically-relevant DDIs in multi-morbid patients under polypharmacy.

## Conclusions

LDL reduction and platelet inhibition remain the cornerstones of cardiovascular prevention in patients at high cardiovascular risk due to a major, acute atherothrombotic event [12]. Therefore, drugs controlling platelet activation and LDL concentrations are essential components of a ‘necessary’ polypharmacy that includes also other agents needed to control those comorbidities that are largely present in CV patients (e.g. hypertension, atrial fibrillation and stressed mental conditions). Polypharmacy and co-morbidities pave the way to the development of clinically relevant and severe DDIs, especially in older adults (Fig. 1). While the clinical impact of the pDDI between ticagrelor and statins deserves further investigation, nevertheless there are sound and plausible pharmacological mechanisms that support caution and careful evaluation when these drugs are given in combination in daily practice. Thus, in specific patient populations at risk of symptomatic DDI, prasugrel can be a valid alternative to ticagrelor in DAPT, since no severe, clinically-relevant DDIs have been found in association with statins [16]. Alternatively, using a lower statin dose in combination with ezetimibe for instance or using bempedoic acid or proprotein convertase subtilisin/kexin type 9 (PCSK9) inhibitors as lipid-lowering monotherapies can also be considered. PCSK9 inhibitors have no PK-based pDDIs [20], but they are not affordable in many countries, while for bempedoic acid more pharmacovigilance data on pDDIs are needed given its relatively recent approval.

## Data Availability

Not applicable for an invited editorial.
